# Unraveling the Binding Mode of TSC2–Rheb through Protein Docking and Simulations

**DOI:** 10.1021/acs.biochem.4c00562

**Published:** 2025-02-13

**Authors:** Berith F. Pape, Shraddha Parate, Leif A. Eriksson, Vibhu Jha

**Affiliations:** † Department of Chemistry and Molecular Biology, University of Gothenburg, Göteborg 405 30, Sweden; ‡ Institute of Cancer Therapeutics, School of Pharmacy and Medical Sciences, Faculty of Life Sciences, University of Bradford, Bradford BD71DP, U.K.

## Abstract

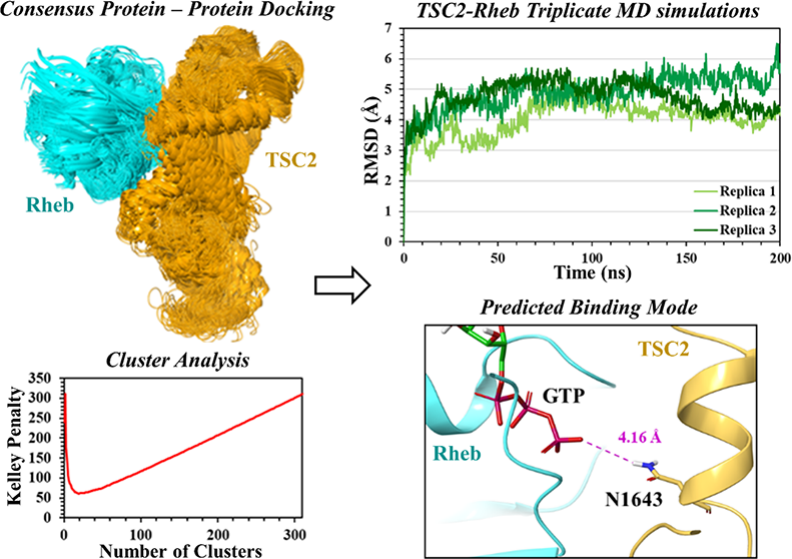

Proteasome inhibitors (PIs) constitute the first line of therapy for multiple myeloma (MM). Despite the impressive clinical efficacy, MM remains fatal due to the development of drug resistance over time. During MM progression, stress responses to hypoxia and PIs suppress mammalian target of rapamycin complex 1 (mTORC1) activity by releasing tuberous sclerosis complex 2 (TSC2), which deactivates Ras homologue enriched in brain (Rheb), a crucial regulator of mTORC1. The efficacy of PIs targeting MM is enhanced when mTORC1 is hyperactivated. We thus propose that the inhibition of TSC2 will improve the efficacy of PIs targeting MM. To the best of our knowledge, no cocrystallized structure of the TSC2–Rheb complex has been reported. We therefore developed a representative model using the individual structures of TSC2 (PDB: 7DL2) and Rheb (PDB: 1XTS). Computational modeling involving an extensive protein–protein docking consensus approach was performed to determine the putative binding mode of TSC2–Rheb. The proposed docking poses were refined, clustered, and evaluated by MD simulations to explore the conformational dynamics and protein mobility, particularly at the drug-binding interface of TSC2–Rheb. Our results agree with the suggested binding mode of TSC2–Rheb previously reported in the literature. The results reported herein establish a basis for the development of new inhibitors blocking the binding of TSC2 and Rheb, aiming to reinstate mTORC1 activation and facilitate improved efficacy of PIs against multiple myeloma.

## 1 Introduction

Multiple myeloma (MM) is one of the most common hematologic malignancies, together with leukemia and non-Hodgkin lymphoma.^1^ Despite the efficacy of existing therapies, such as proteasome inhibitors (PIs), a major problem is the development of resistance, resulting in relapse in most patients.^2,3^ Consequently, there is a significant unmet medical need for novel strategies to improve patient outcomes.

Stress is inevitably encountered in cancer cells, and tumors can only grow if they adapt to these changes. The integrated stress response (ISR) is one of the pathways that cancer cells activate to avoid cell death.^4^ ISR is a set of signaling pathways that regulate cellular responses to various factors such as ER stress, hypoxia, oxidative stress, and nutrient deficiency, and thus helps to restore cellular homeostasis.^5,6^ The ISR stress signals are transmitted via four serine/threonine kinases: GCN2, HRI, PERK, and PKR, which all phosphorylate the translation initiation factor. Cancer is often treated with therapeutic agents acting on the integrated stress response. In MM, proteasome inhibitors block the function of the proteasome, causing proteotoxicity, which results in ER stress and leads to cell death.^7^ Darawshi et al. showed that the stress caused by both PIs and hypoxia triggered cells *in vitro* to activate the kinase HRI, which enables a negative feedback response to suppress mTORC1 activity.^8^ By hyper-activating mTORC1, toxicity to PIs was reinstated in MM cells.^8^ Due to the constitutive activation of mTORC1, protein synthesis is initiated, and this excessive demand, in combination with PIs, results in proteotoxicity and subsequent cell death. For that reason, enforcing the activity of mTORC1 in combination with PIs may be an important strategy to treat MM.

mTORC1 is a serine/threonine protein kinase that belongs to the PI3K-related kinases. mTOR is the master regulator in the PI3K-Akt-mTOR pathway that controls vital cellular processes such as the regulation of the cell cycle, metabolism, and protein synthesis.^9^ The upstream pathway of mTORC1 involves the tuberous sclerosis complex (TSC) and the small GTPase Rheb.^14^ GTPases function as molecular switches and regulate different intracellular processes.^10,11^ Rheb is part of the Ras superfamily, which all share the common phenomenon that conformational changes take place while binding to GTP and GDP.^12,13^ It has been well established that Rheb is essential for the activation of mTORC1 and its downstream pathways.^14,16−18^ The activity of Rheb depends on its nucleotide-binding state and activates mTORC1 in its active GTP-bound form. The TSC complex, the GAP protein of Rheb,^14,15^ in turn, suppresses mTORC1 by inactivation of Rheb.^16,17^ As shown by Darawshi et al., reactivation of mTORC1 by suppressing the TSC complex reinforces the toxicity of PIs targeting MM.^8^

An important aspect to consider in the multiple myeloma (MM) treatment strategy is the loss-of-function mutations in the TSC complex resulting in a genetic disorder, tuberous sclerosis complex (TSC), also known as tuberous sclerosis. This genetic disorder is marked by the growth of nonmalignant tumors in multiple organs, including the brain, skin, kidneys, and heart. Despite the occurrence of TSC, targeting the inhibition of the TSC2 complex holds significant potential for advancing drug development efforts aimed at treating multiple myeloma (MM) because multiple myeloma (MM) is a life-threatening malignancy with an urgent need for immediate and aggressive treatment. The therapeutic advantages of inhibiting TSC2, i.e., exploiting the cancer cell dependence on mTORC1 signaling, may surpass the potential risks associated with inducing a chronic but nonfatal condition, TSC. Furthermore, it is highly probable that adverse effects analogous to those seen in TSC disease can be effectively mitigated through the implementation of combination therapies.

**Figure 1:**
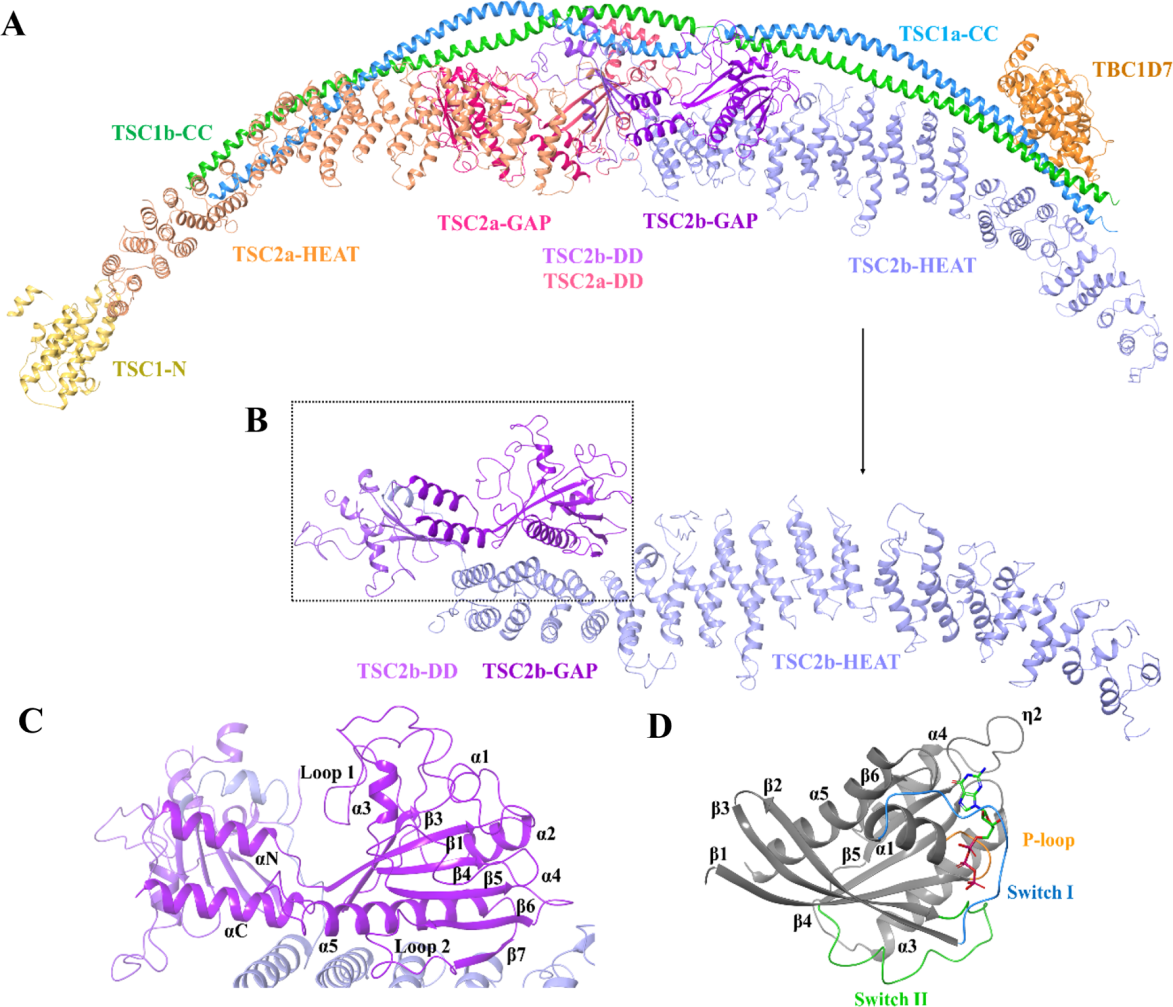
Cartoon representation of (A) TSC (PDB: 7DL2) – subunits TSC1a-CC (blue), TSC1b-CC (green), TSC1-N (yellow), TSC2a-HEAT (light orange), TSC2b-HEAT (light blue), TSC2a-GAP (magenta), TSC2b-GAP (purple), TSC2a-DD (light magenta), TSC2b-DD (light purple), and TBC1D7 (orange). (B) TSC2 Chain A (purple). (C) Close-up view of the TSC2-GAP region (purple). (D) Rheb (gray, PDB: 1XTS) with GTP in stick model (green). Secondary structure elements in (C–D) are labeled. Switch I, Switch II, and the P-loop in (D) are colored in blue, green, and orange, respectively.

The TSC complex entails the subunits TSC1, TSC2, and TBC1D7 in a 2:2:1 stoichiometric quantity (Figure 1A).^19,26^ As the GAP protein of Rheb, the TSC complex catalyzes the formation of the inactive conformation of Rheb by the hydrolysis of GTP to GDP in the TSC2 GAP domain^15,17^ (Figure 1C).

The hydrolysis proceeds through nucleophilic attack on the γ-phosphate group of GTP by a water molecule, producing GDP by cleavage of the phosphate monoester bond.^19,20^ The mechanism of hydrolysis differs depending on the type of GTPase, with the arginine-finger GAPs (Ras and Rho GTPases) being the most well-known.^21^ During hydrolysis, this finger positions a glutamine residue of the GTPase to activate a water molecule for the nucleophilic attack, and both amino acid residues stabilize negative charges in the transition state.^10^ Although Rheb is most closely related to the small GTPases H-Ras and Rap2,^21^ it displays, based on models and mutational studies, some major differences in its molecular function as a result of variations in key amino acids.^12,22^ Furthermore, based on modeling and mutational insights, the TSC complex does not contain the arginine finger as used by Ras and Rho GAPs, but instead has an asparagine-thumb GAP domain (Asn1643)^12,22^ (Figure S1). The use of an Asn-thumb in TSC2 to promote GTP hydrolysis has been established through mutational analysis, confirming that the residue is positioned ideally to form a hydrogen bond with the water molecule engaged in the nucleophilic attack of the GTP γ-phosphate.^22^ Simultaneously, the hydroxyl group of Y35^Rheb^ assists the positioning of GTP by the formation of a hydrogen bond with the γ-phosphate of GTP^23^ (Figure S2). One of the residues, Q64^Rheb^, is equivalent to Q61^Ras^. Q61^Ras^ is essential in the GTP hydrolysis, whereas Q64^Rheb^ cannot contribute to GTP hydrolysis as it is sterically blocked.^12,23^ Instead, it is confirmed by mutagenesis studies that D65^Rheb^ indirectly plays a key role in GTP hydrolysis^12^ (Figure S2). D65 ^Rheb^ stabilizes the TSC2–Rheb complex by interactions with residues G1595^TSC2^ and G1596 ^TSC2^ (located on loop 1, Figure S2). These residues differ in sequence compared with Rap1GAP and could therefore render selectivity. Together with D65^Rheb^, R15^Rheb^ is one of the key amino acid residues. This arginine is conserved in the position similar to that of G12^Ras^. Mutational studies of G12^Ras^ revealed reduced activity of Ras and severely decreased ability of RasGAPs to promote GTP hydrolysis, as the mutations would interfere with requirements for the transition state.^24^ In contrast, mutations in R15^Rheb^ do not exhibit a different GTP loading.^22^ This again suggests that TSC2–Rheb utilizes another mechanism. R15^Rheb^ supports the catalysis indirectly by stabilizing the active conformation through formation of salt bridges with D1636^TSC2^ and H1640^TSC2^ positioned on the catalytic helix^12^ (Figure S1). Mutations of H1640^TSC2^ likewise confirmed that it is essential for the GAP activity. Biochemical studies have mapped out additional residues to be essential for TSC2-GAP activity.^22,23^ F1666^TSC2^, which is part of the hydrophobic binding site of TSC2-GAP located beside the catalytic helix α3 (Figure S2), interacts with residues P37^Rheb^ and I39^Rheb^ located on switch I (Figure S1). K1638^TSC2^ stabilizes the catalytic α3-helix by the formation of an intramolecular salt bridge with E1558^TSC2^ (Figure S1).^12^

To the best of our knowledge, no cocrystallized structure of Rheb bound to the TSC2 complex has been solved yet. Such data are needed for a thorough understanding of the mechanism of action and how Rheb binds to TSC2. In this work, we have performed extensive protein–protein docking to propose a suitable model that can rationalize the protein–protein interactions between the TSC2-GAP domain and the Rheb catalytic binding site. In-depth analyses of structures, molecular dynamics (MD) simulations, and MM-GBSA binding free energy calculations allowed us to gain structural insights into the protein–protein interactions between TSC2 and Rheb that lead to the hydrolysis of GTP to GDP and the subsequent inactivation of Rheb. Furthermore, the best TSC2–Rheb model identified from our computational protocol can be used as a template for the design of TSC inhibitors. The contact between TSC2 and Rheb is limited, and it is, therefore, feasible that small molecules could disrupt this protein–protein interaction. Consequently, this would be an attractive strategy for the design of inhibitors that activate mTORC1 and reinstate the toxicity of proteasome inhibitors in MM.

## 2 Materials and Methods

### 2.1 Selection and Preparation of TSC2 (Cryo-EM) and Rheb (X-Ray) Structures

TSC2-GAP is the catalytic subunit of the TSC complex, where Rheb binds. It was decided to truncate the TSC structure and eliminate chains that are not directly involved in the catalytic reaction. In order to obtain the most reliable model of the TSC2–Rheb complex, the structure of TSC2 was truncated in three distinct stages to obtain a more thorough understanding of how Rheb is positioned in the binding site of TSC2. The protein preparation wizard tool^25^ implemented in Schrödinger was used to prepare the structures of TSC2-GAP (PDB: 7DL2)^26^ and the small human GTPase Rheb in complex with GTP (PDB: 1XTS).^27^ The three truncated TSC2 models were obtained by deleting the secondary structures of certain parts of the complex. The residues retained in the structures are complex 1 (1537–1730), complex 2 (1015–1071, 1195–1210, and 1524–1755), and complex 3 (1015–1082, 1182–1245, and 1495–1755). Hydrogen atoms were added, and missing loops were generated using the Prime module^28^ of Schrödinger.^25^ Protonation and tautomeric states of Asp, Glu, Arg, Lys, and His residues were determined at pH 7.4. Finally, the OPLS4 force field^29^ was applied during restrained minimization of Rheb and the truncated TSC2 structures, respectively, to refine the protein geometries.

### 2.2 Protein–Protein Docking

**Figure 2:**
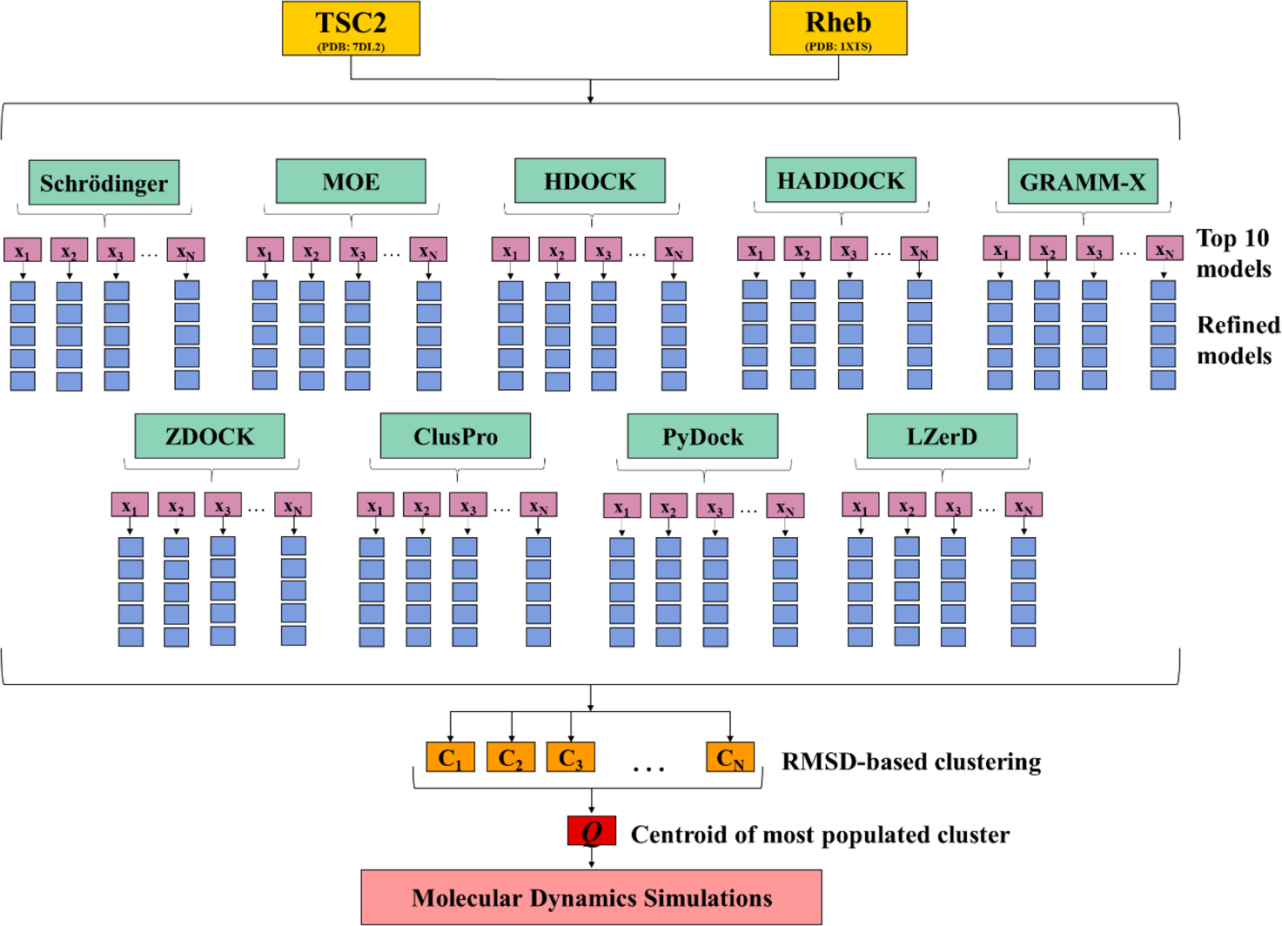
Schematic representation of the protein–protein docking strategy used for the prediction of the TSC2–Rheb complex by employing nine different protein–protein docking engines (Piper (Schrödinger), MOE, HDOCK, HADDOCK, GRAMM-X, ZDOCK, ClusPro, PyDock, and LZerD).

To generate the most accurate model of the TSC2–Rheb complex, we employed our previously designed consensus strategy,^30^ combining the benefits of nine of the most employed protein–protein docking engines: Piper (Schrödinger),^31^ MOE,^32^ HADDOCK,^33,34^ ClusPro,^35,36^ HDOCK,^37,38^ LZerD,^39,40^ ZDOCK,^41,42^ PyDockWeb,^43^ and GRAMM.^44,45^ A schematic representation of the process used is shown in Figure 2. In all the above-mentioned protein–protein docking engines, Rheb was used as a ligand (smaller protein) to be docked onto TSC2 (larger protein). The protein–protein interaction interface at the catalytic site has been predicted by Hansmann et al.^12^ and after visual inspection, the residues G1595, G1596, R1634, D1636, R1639, H1640, and F1666 of TSC2 and R15, P37, I39, D65, and T88 of Rheb were defined as interacting residues and used as restraints with a distance of 2–10 Å as lower and upper limits, respectively, in all docking engines. The top ten predicted complexes from each docking engine were refined by means of the GalaxyRefineComplex tool using default settings.^46^ For each docking pose, the five lowest refined energy complexes were used as the final models. The obtained refined complexes were clustered based on RMSD values of all heavy atoms using the “Clustering of Conformer” model implemented in Maestro, Schrödinger.^47^ Each model hereafter has the following name, X-m-n, and represents the refined model *n* (*n* = 1–5) from the predicted model *m* (*m* = 1–10) obtained from the docking engine X. The optimum number of clusters was determined from Kelley penalty plots.^48^ The model nearest the centroid of the most populated cluster in the optimal distribution was employed as the final docking pose, *Q*. This strategy was applied for each of the three TSC2–Rheb complexes, resulting in three final structures, *Q1*, *Q2,* and *Q3*, further subjected to classical MD simulations.

### 2.3 MD Simulations and Trajectory Clustering

a. Systems preparation: The systems include the predicted TSC2–Rheb complexes (Section 2.2) in which TSC2 is truncated in three different ways (complexes *Q1, Q2, and Q3*). The systems were prepared as discussed in Section 2.1.
b. Molecular dynamics simulation protocol: All molecular dynamics (MD) simulations of *Q1, Q2*, and *Q3* were carried out for 200 ns using the Desmond MD simulator engine within default settings,^47^ as implemented in Schrödinger. The NPT ensemble was used for minimization and relaxation of each system and the OPLS4 force field^29^ applied during all simulations. Each protein–protein complex was solvated in a TIP3P water model. Periodic boundary conditions were applied in all directions along with a 10 Å water buffer around the protein complex in an orthorhombic simulation box.^49^ The overall charge of the system was balanced by Na^+^/Cl^–^ counterions, and the salt concentration was set to 0.15 M to reproduce physiological conditions. In all simulations, the temperature and pressure of the system were maintained constant at 300 K and 1 atm bar atmospheric pressure, regulated by the Nose-Hoover thermostat^50^ with a relaxation time of 1 ps, and the Martyna-Tobias-Klein barostat^51^ with a relaxation time of 2 ps and isotropic coupling, respectively. Nonbonded forces were calculated using an r-RESPA integrator and the long-range electrostatic interactions were calculated using the particle mesh Ewald method.^52^ The simulations of the TSC2–Rheb model *Q3* was run in an additional set of triplicates (referred to as replica 1, 2, and 3), and in addition the TSC2 model in *Q3* was simulated alone for 1000 ns, to explore its stability and dynamics prior to Rheb binding.
c. MD simulation trajectories were analyzed for root-mean-square deviations (RMSD) and root-mean-square fluctuations (RMSF) of the protein–protein complexes using the “simulation interaction diagram” module in Schrödinger. The obtained trajectories were clustered according to RMSD using the “Desmond Trajectory Clustering” module^47^ setting up a frequency value of 10 (every 10th ns) and up to a maximum of 10 clusters, to finally select the representative structure from the most populated cluster.

### 2.4 MM-GBSA Binding Free Energy Calculations

The protein–protein binding energies were computed using the Prime MM-GBSA module^28^ in Schrödinger with the OPLS4 force field^29^ and VSGB solvation model.^53^ MM-GBSA free energies of binding (Δ*G*_bind_) were calculated for the representative MD trajectory structures using the equation:
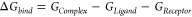
1where Δ*G*_*bind*_ is the total binding free energy of binding (TSC2 as the receptor and Rheb as the ligand). Δ*G*_*Complex*_, *ΔG,*_*Ligand*_, and *ΔG*_*Receptor*_ represent the energy calculations carried out in the Prime MM-GBSA module for the optimized complex, optimized free ligand, and optimized free receptor, respectively. Frames were extracted every 2 ns of the MD simulations to obtain an average *ΔG*_*bind*_.

### 2.5 Data and Software Availability

The protein crystal structures were downloaded from the Protein Data Bank, https://www.rcsb.org/. Protein–protein docking was performed using the open-source web servers HADDOCK https://wenmr.science.uu.nl/haddock2.4/, ClusPro https://cluspro.org/home.php, ZDOCK https://zdock.umassmed.edu/, HDOCK http://hdock.phys.hust.edu.cn/, LZerD https://lzerd.kiharalab.org/about/, GRAMM-X http://gramm.compbio.ku.edu/gramm, and PyDockWeb https://life.bsc.es/pid/pydockweb, using default settings unless indicated otherwise in the text. In addition, MOE 2022–02 (paid license, https://www.chemcomp.com/index.htm) was used for protein–protein docking, and Schrödinger 2022–3 (PIPER, www.schrodinger.com; paid license) was used for protein–protein docking. The Schrödinger software was also used for complex clustering, protein structure, MD simulations, alanine scanning, and MM-GBSA energies. Complex refinements were performed using the web server GalaxyWeb https://galaxy.seoklab.org/cgi-bin/submit.cgi?type=COMPLEX. Alanine scanning was performed using the web servers DrugScorePPI https://cpclab.uni-duesseldorf.de/dsppi/main.php and BUDE alanine scanning https://pragmaticproteindesign.bio.ed.ac.uk/balas/.

## 3 Results and Discussions

### 3.1 Protein–Protein Docking Analyses

**Figure 3:**
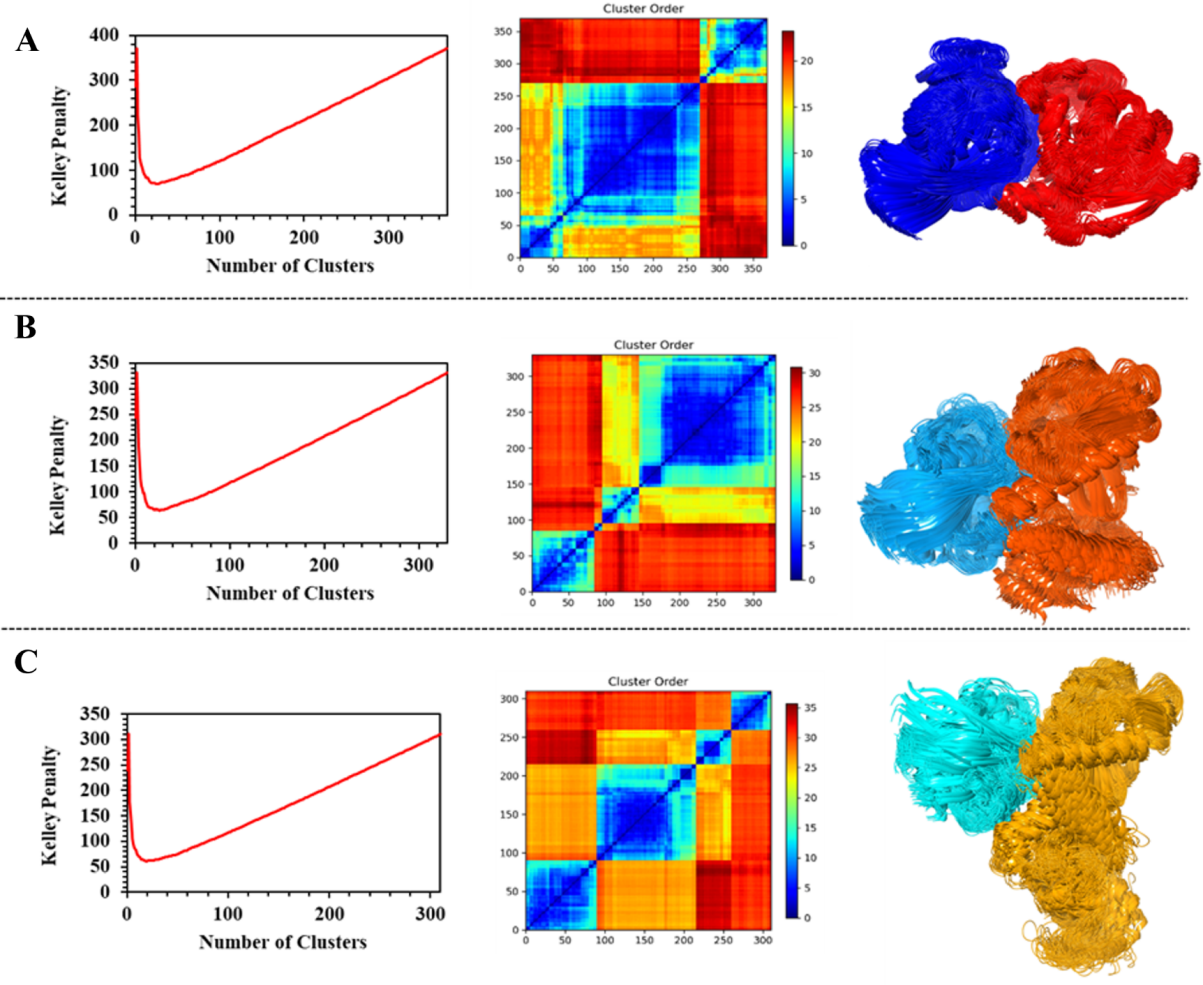
Structural data from the clustering of TSC2-Rheb complexes. Kelley penalty plot, distance matrix, and most populated cluster with (A) 129 members from the docking of complex 1 (TSC2: red, Rheb: navy blue); (B) 92 members from the docking of complex 2 (TSC2: orange, Rheb: aqua); (C) 70 members from the docking of Complex 3 (TSC2: yellow, Rheb: turquoise).

As discussed in Section 2.1, no X-ray crystal or Cryo-EM structure of Rheb bound to TSC2 is available. Employing a consensus docking approach using nine of the most widely used protein–protein docking engines: Piper (Schrödinger),^31^ MOE,^32^ HADDOCK,^33,34^ ClusPro,^35,36^ HDOCK,^37,38^ LZerD,^39,40^ ZDOCK,^41,42^ PyDockWeb,^43^ and GRAMM,^44,45^ as per the schematic representation in Figure 2, the top ten (or stated otherwise) predicted complexes from each docking engine (Table S1) were selected for refinement. The refinement protocol allowed for improved model accuracy, providing flexibility at the protein–protein interface, and reporting of conformational changes taking place upon binding.^46^ The refined models were furthermore subjected to RMSD-based conformation clustering. According to the Kelley penalty plot, the results of conformation clustering indicated the optimum number of clusters for complexes 1, 2, and 3 were 25, 26, and 19, respectively, along with the associated distance matrix as shown in Figure 3. The most populated clusters of the TSC2-Rheb complexes 1, 2, and 3 contained 129, 92, and 70 structures, respectively. The models closest to the centroid of the most populated cluster for each complex (*Q1*, *Q2,* and *Q3*) are depicted in Figure S3.

The contribution of each docking engine in the most populated cluster of each complex is shown in Figure S4. The most populated cluster for complex 1 comprises 129 members (Figure 3A) to which LZerD, HDOCK, HADDOCK, ClusPro, and ZDOCK contribute with 39%, 27%, 22%, 8%, and 4% of the docking poses, respectively. The model closest to the centroid is *LZerD-7–5*. Complex 2 has the most populated cluster consisting of 92 members (Figure 3B), to which the docking engines HADDOCK, HDOCK, and ClusPro contribute with 54%, 29%, and 16% of the poses, respectively. The model closest to the centroid is *HDOCK-2–2*. Finally, complex 3 has the most populated cluster of 70 members (Figure 3C), divided between the docking poses from HDOCK, HADDOCK, and ClusPro, which contribute with 57%, 36%, and 7% thereof, respectively. The model closest to the centroid is *HDOCK-1–3*. Taken together, it was found that HDOCK, HADDOCK, and ClusPro are the docking engines that contribute largely to the most populated cluster in each protein–protein complex. Moreover, HDOCK and HADDOCK together contribute at least 49% of the models present in each of the most populated clusters. The model closest to the centroid in two of the three complexes is derived from the HDOCK docking engine.

**Figure 4:**
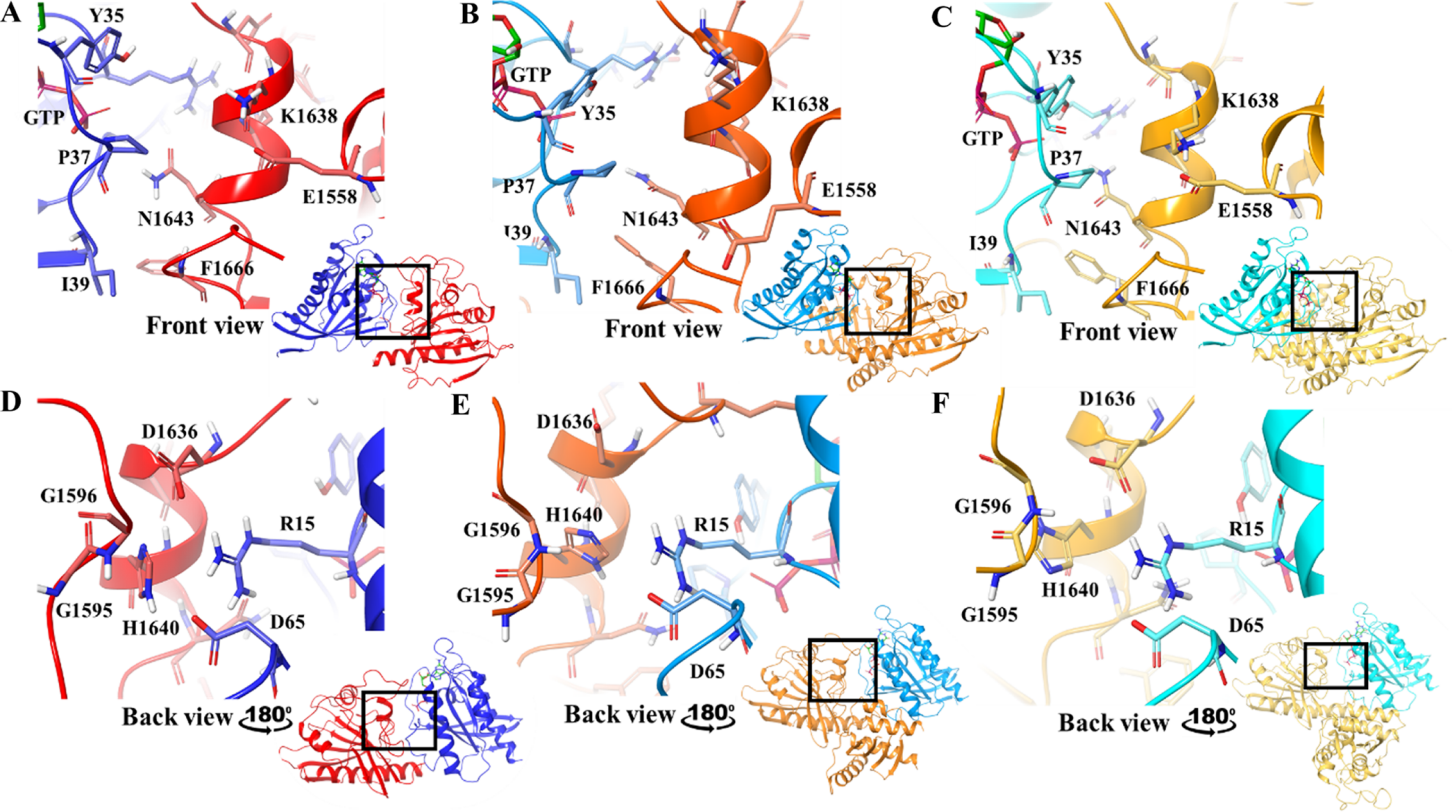
Close-up view of the binding site with Y35, P37 and I39 (Rheb), and E1558, K1638 and N1643 (TSC2) shown as sticks: (A) Q1 (Rheb: navy blue, TSC2: red) (B) Q2 (Rheb: aqua, TSC2: orange) (C) Q3 (Rheb: turquoise, TSC2: yellow). Rotated view of the binding site with R15 and D65 (Rheb), and G1595, G1596, D1636 and H1640 (TSC2) shown as sticks: (D) Q1 (Rheb: navy blue, TSC2: red), (E) Q2 (Rheb: aqua, TSC2: orange) and (**F**) Q3 (Rheb: turquoise, TSC2: yellow).

The models closest to the centroid of the most populated cluster – *LZerD-7–5*, *HDOCK-2–2,* and *HDOCK-1–3,* which represent complexes *Q1*, *Q2,* and *Q3*, respectively, were selected and superposed by the “Protein Structure Alignment Tool” implemented in Schrödinger Maestro.^25^ Superposition of the three docking poses demonstrates that Rheb is positioned slightly differently in *Q1* as compared to *Q2* and *Q3* (Figure S5). The smaller model of TSC2 used in *Q1,* in which the helices αN and αC (Figure 1C) are not present, makes space for the α3 and α4 helices of Rheb to be oriented slightly more toward the catalytic α3 helix of TSC2. However, despite structural differences between *Q1*, *Q2,* and *Q3*, the binding sites remain quite similar. As reported by Marshall et al.,^22^ Mazhab-Jafari et al.,^23^ and Hansmann et al.,^12^ the residues N1643, K1638, E1558, G1595, G1596, H1640, D1636, and F1666 at the catalytic α3 helix of TSC2 and the residues R15, Y35, P37, I39, and D65 at switch I and II of Rheb (Figure 4) are placed at the TSC2–Rheb interface and thus considered as key residues for binding recognition. Our docked models were visually compared with the model of the catalytic site predicted by Hansmann et al.^12^ (Figure S6) and found to be comparable, with Rheb and its key residues positioned in similar orientations.

### 3.2 MD Simulations of *Q1*, *Q2,* and *Q3*

**Figure 5:**
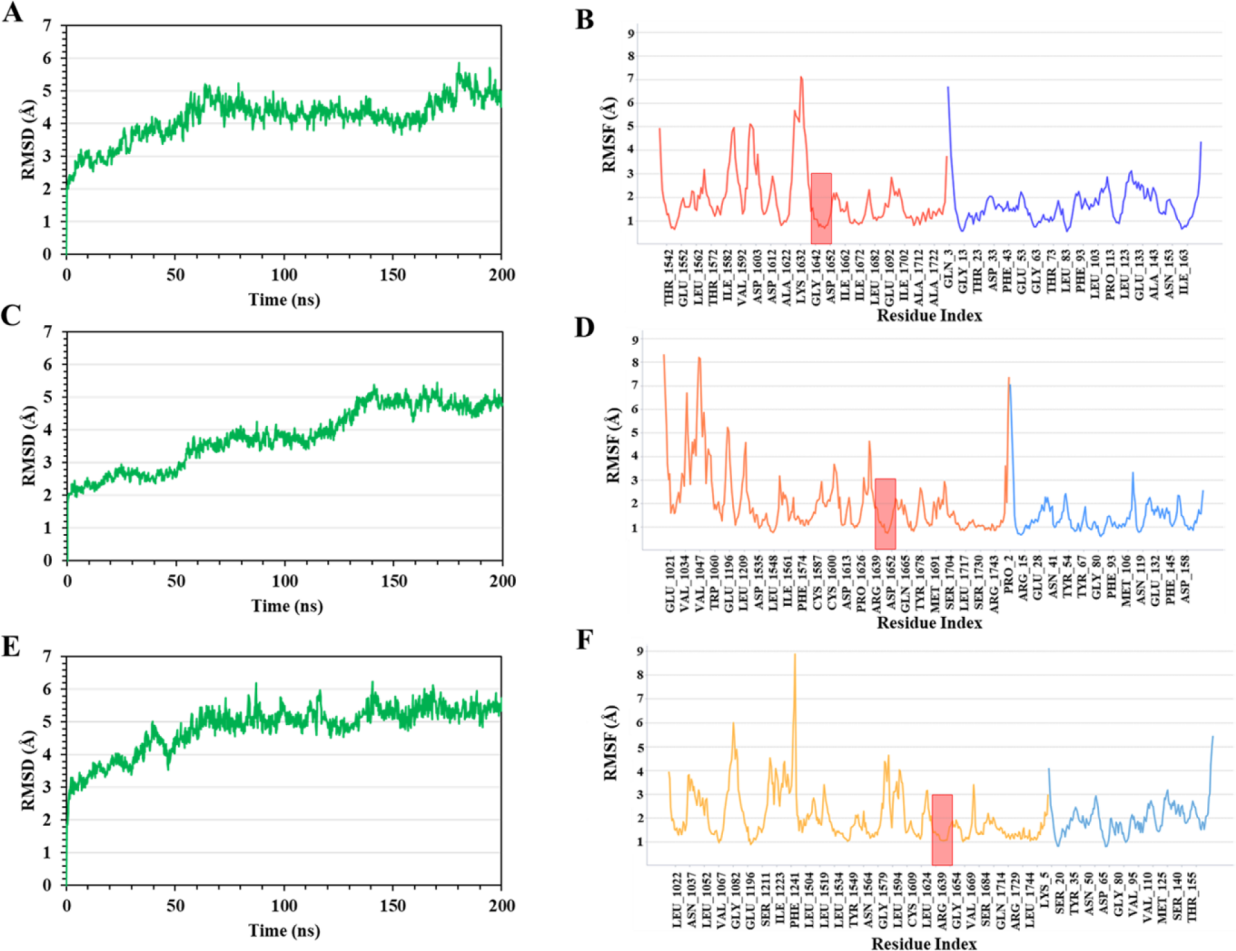
α-carbon RMSD plot of the TSC2-Rheb models (A) *Q1*, (C) *Q2,* and (E) *Q3* during the 200 ns MD simulations. RMSF plot of the models (B) *Q1,* (D) *Q2,* and (F) *Q3* during 200 ns MD simulations. The red area in the RMSF plots indicates the residues of the binding site in the catalytic helix α3 of TSC2.

Since conventional docking programs only consider rigid binding poses of the protein–protein complexes, MD simulation is one of the most widely used postdocking techniques in order to take into account the flexibility and dynamic nature of proteins. The conformational stability of each system (*Q1*, *Q2,* and *Q3*) was analyzed by performing 200 ns MD simulations as described in Section 2.3. To assess the dynamic stabilities of the three complexes, the evolution of the root-mean-square deviation (RMSD) with respect to the initial minimized and equilibrated structure was calculated (Figure 5). The RMSD values were analyzed as functions of simulation time. The MD simulations of *Q1* (Figure 5A) revealed that the system appears stable, as demonstrated by low and constant RMSD values over the course of the simulation, showing convergence within 70 ns. In comparison with *Q1*, the initial protein–protein complex of *Q2* (Figure 5C) seems to be less stable, as evidenced by the trajectory in which the convergence was not reached until 140 ns of the 200 ns simulation trajectory. The MD simulation of *Q3* (Figure 5E) is characterized by a similar pattern as *Q1*, converging within 60 ns and showing no major fluctuations during the simulation trajectory.

In addition to RMSD values, we also calculated root-mean-square fluctuations (RMSF) of each TSC2-Rheb residue in the three systems (*Q1*, *Q2,* and *Q3*) throughout the 200 ns simulations (Figure 5). The protein structures share similar RMSF distributions and similar trends in their dynamic features, with the major fluctuation occurring in the terminal residues and the loop regions. In particular, residues G1021–T1060 of *Q2* (Figure 5D) were found to fluctuate significantly, showing RMSF values reaching ∼8 Å. The binding site region is stable around the catalytic α3 helix (C1635–F1645, red area of Figure 5D); however, the loop region prior to the α3 helix (A1622–R1634) shows higher RMSF values, reaching ∼4.5 Å. Similarly, model *Q1* shows an RMSF below 2 Å for the α3 helix, but the preceding loop region displays high RMSF values (Figure 5B). In contrast to *Q1* and *Q2*, in the *Q3* model, the RMSF values are lower for both the α3 helix and the loop region, thus showing higher stability (Figure 5F).

In order to gain further insights into the protein–protein interactions, the binding free energies were calculated every 2 ns of the MD trajectory for the three models using the MM-GBSA method. The average binding free energies (Δ*G*_bind_) of models *Q1*, *Q2,* and *Q3*, along with the contribution of van der Waals energy (Δ*G*_vdW_), lipophilic energy (Δ*G*_Lipo_) and hydrogen bond energy (Δ*G*_Hbond_) are listed in Table 1. As observed, model *Q2* produced the highest Δ*G*_bind_ value of −119 kcal/mol, relative to the −99 and −106 kcal/mol Δ*G*_bind_ values for *Q1* and *Q3*, respectively. Taken together, the MM-GBSA energy properties suggest that model *Q2* presents the most favorable binding mode of TSC2-Rheb; however, all different free energy components are in close proximity between the three models. The close range between the models demonstrates the reliability of the consensus protein–protein docking protocol employed in the study.

**Table 1:** MM-GBSA Binding Free Energy for the MD Trajectories of *Q1*, *Q2* and *Q3* in kcal/mol with Corresponding Standard Errors

Entry	Δ*G*_bind_	Δ*G*_vdW_	Δ*G*_Hbond_	Δ*G*_Lipo_
*Q*1	–99 ± 13	–103 ± 10	–7.0 ± 1.9	–27 ± 3.1
*Q*2	–119 ± 18	–122 ± 14	–9.4 ± 1.9	–27 ± 4.0
*Q*3	–106 ± 21	–115 ± 19	–9.7 ± 1.7	–23 ± 4.1

Based on the binding free energy calculations, MD analyses, and visual inspection, it was concluded that the largest model *Q3* is the most stable and promising model, giving a plausible binding mode of TSC2–Rheb. Furthermore, the stability of *Q3* makes us believe that the binding mode would not display any major variations when taking into account the full protein structure. We thus decided to proceed with this model in the subsequent steps.

### 3.3 Triplicate MD Simulations of *Q3*

**Figure 6:**
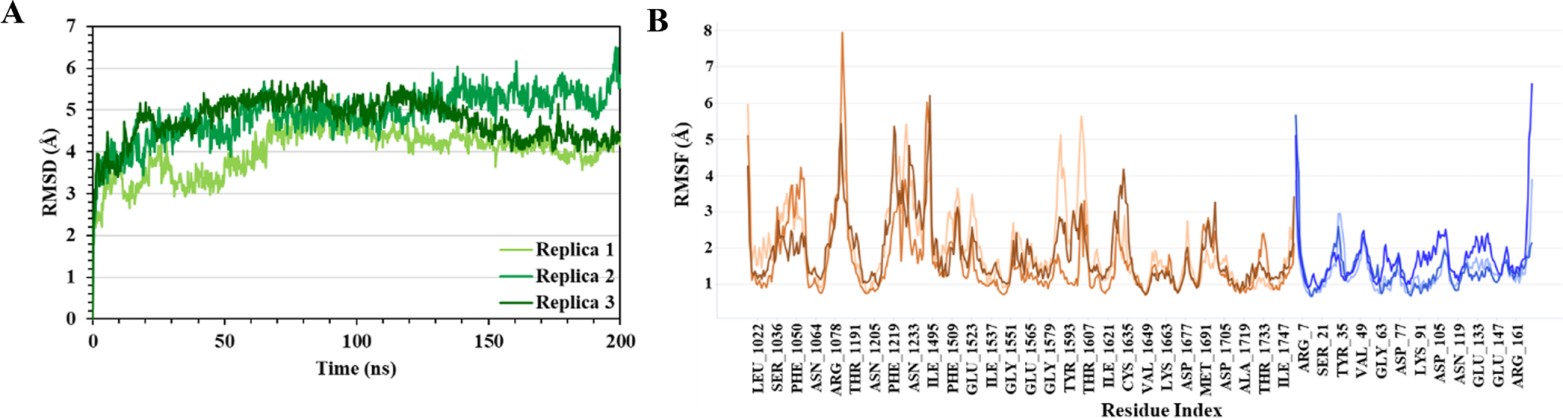
Triplicate MD simulations of TSC2-Rheb model *Q3*. (A) α-carbon RMSD plot. (**B**) RMSF plots. Brown colors represent TSC2 (light: Replica 1, medium: Replica 2, dark: Replica 3), blue colors represent Rheb (light: Replica 1, medium: Replica 2, dark: Replica 3).

To validate the structural robustness and dynamic stability of *Q3* and to gain deeper insights into the binding site, model *Q3* was analyzed by additional triplicate MD simulations, with each simulation having a duration of 200 ns, as described in Section 2.3. The triplicate simulations revealed that the *Q3* protein–protein complex is stable with low and constant RMSD values, reaching convergence within 60 ns (Figure 6A). Furthermore, there were no major variations between the simulation trajectories, as observed from the RMSD plots. Analysis of RMSF versus the residue number is given for each simulation in Figure 6B. The simulations share similar RMSF distributions and trends in their dynamic features. The binding site region of TSC2 (I1621–V1649) is relatively rigid, with fluctuations primarily in the loop region thereof (I1621–C1635). The major movements occur, as anticipated, in the terminal residues and loop regions.

The binding free energy was calculated every 2 ns of the MD trajectory by using the MM-GBSA method, resulting in 100 frames for each simulation (Table 2 and Figure S7). The average values were calculated over the total simulation time, as described in Section 2.4. Replica 3 demonstrated to be the most stable conformation of the TSC2–Rheb complex. Binding free energy (Δ*G*_bind_), van der Waals energy contribution (Δ*G*_vdW_), hydrogen bond energy contribution (Δ*G*_Hbond_) and lipophilic energy contribution (Δ*G*_Lipo_) were found to be −149 kcal/mol, −150 kcal/mol, −8.9 kcal/mol, and −28 kcal/mol, respectively, for Replica 3, notably better than the other two replicas.

**Table 2:** MM-GBSA Binding Free Energy for the MD Trajectories of replica 0, 1, 2, and 3 in kcal/mol with Corresponding Standard Errors

Entry	Δ*G*_bind_	Δ*G*_vdW_	Δ*G*_Hbond_	Δ*G*_Lipo_
Replica 0	–106 ± 21	–115 ± 19	–9.7 ± 1.7	–23 ± 4.1
Replica 1	–97 ± 18	–121 ± 13	–5.8 ± 1.8	–27 ± 3.5
Replica 2	–121 ± 25	–121 ± 18	–7.7 ± 1.9	–29 ± 4.5
Replica 3	–149 ± 13	–150 ± 9.0	–8.9 ± 1.3	–28 ± 2.5
Average	–118 ± 19	–126 ± 14	8.0 ± 1.7	–27 ± 3.7

**Figure 7:**
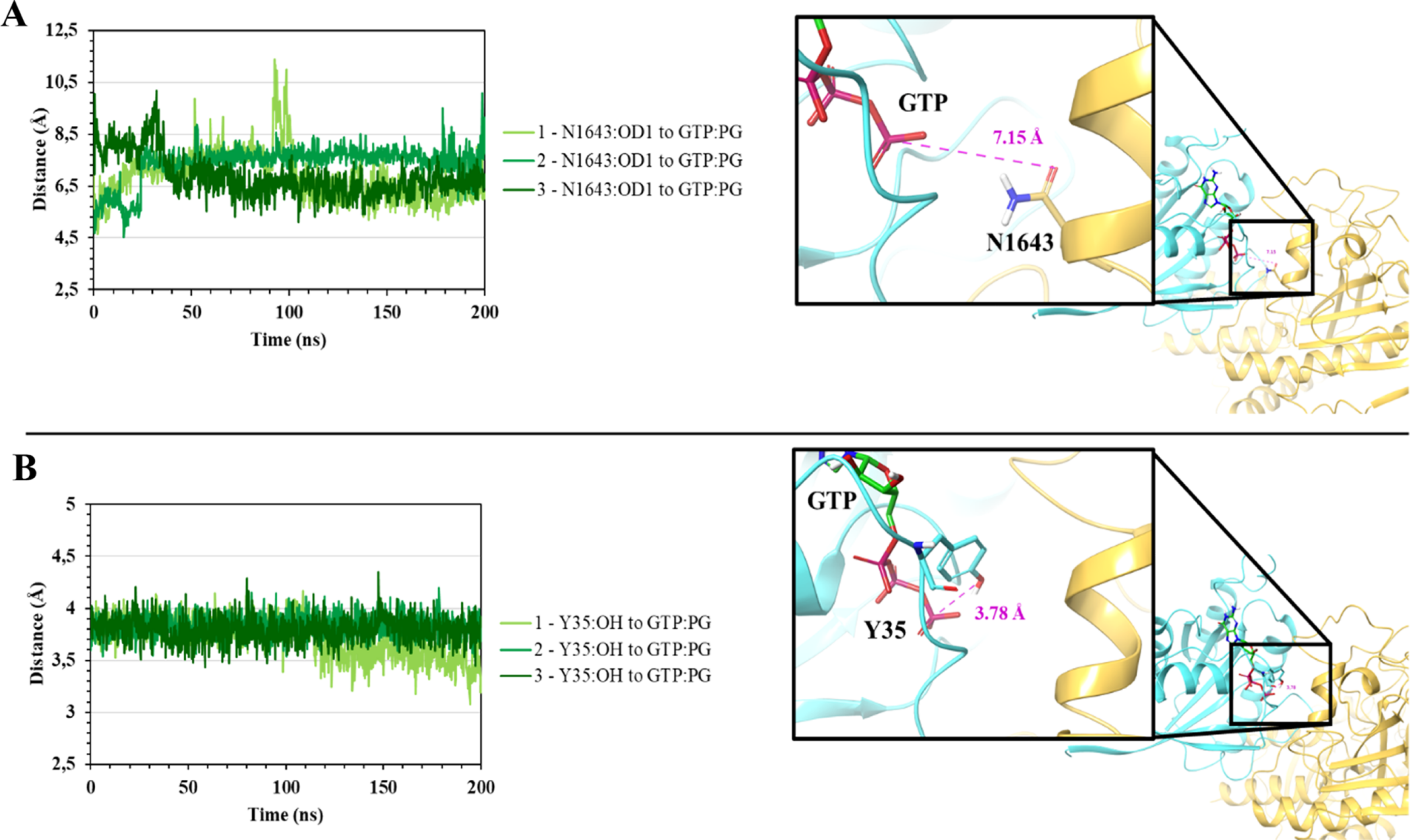
(**A**) Distance between N1643^TSC2^ and GTP, water molecule hidden. (**B**) Distance between Y35^Rheb^ and GTP. TSC2 and Rheb protein ribbons are colored in golden and cyan, respectively, while the phosphate group of GTP is shown in red.

Another aspect that remains essential to analyze is the capability of the TSC complex to promote the hydrolysis of the bound GTP molecule in Rheb to GDP. In the TSC2-GAP region, the asparagine thumb N1643^TSC2^ positions a water molecule for the nucleophilic attack on GTP.^10^ To validate the possibility of hydrolysis occurrence, the distance (Å) between N1643^TSC2^ and the γ-phosphate group was calculated throughout the triplicate simulations. As shown in Figure 7A, the distance exhibits only minimal fluctuations, with an average of 7.0 ± 0.8 Å. Therefore, N1643^TSC2^ is in an acceptable range to position a water molecule for the nucleophilic attack of the γ-phosphate group of GTP. Furthermore, Y35^Rheb^ assists the positioning of GTP by the formation of a hydrogen bond with the γ-phosphate of GTP.^24^ In Figure 7B, the distance between the hydroxyl group of Y35^Rheb^ and the γ-phosphate of GTP is given. The distances demonstrate stable H-bonding between the two, with an average distance of 3.79 ± 0.1 Å for all three replicas.

### 3.4 Residue Scanning and Interaction Analysis

The key residues of *Q3* taking part in the binding recognition of TSC2–Rheb were analyzed using the residue scanning technique. The purpose of alanine scanning is to detect the individual contribution of a small set of residues (also called binding hotspots) that influence the binding free energy between two protein structures. A binding hotspot is a residue that causes a significant decrease in binding free energy when mutated to an alanine.^54^ When small molecules target these hotspots, they can act as effective inhibitors of protein–protein interactions (PPIs). The interface residues between TSC2 and Rheb in complex *Q3* were thus scanned using the “Residue Scanning Calculations” implemented in BioLuminate, Schrödinger,^55^ as well as two web servers: DrugScorePPI^56^ and BUDE Alanine Scan.^57,58^ The residues of TSC2 demonstrating the largest decrease in binding affinity are listed in Table 3.

**Table 3:** Summary of Results from Alanine Scanning of TSC2 Interface Residues in Model *Q3*

	Change in binding free energy Δ*G*_bind_ (kcal/mol)		
Residues	BioLuminate	DrugScorePPI	BUDE Alanine scan
R1529A	10.17	0.80	2.6
L1533A	1.79	0.73	3.9
I1537A	3.97	0.40	2.7
H1633A	4.80	0.16	4.0
R1634A	11.81	1.84	7.8
D1636A	0.69	1.36	5.8
R1639A	12.14	1.82	6.6
Q1665A	5.91	0.10	7.8
F1666A	7.51	0.68	7.3
T1733A	9.92	0.55	7.3
I1735A	6.47	1.17	5.3
R1749A	25.33	1.54	8.3
R1753A	12.61	1.69	4.5

**Figure 8:**
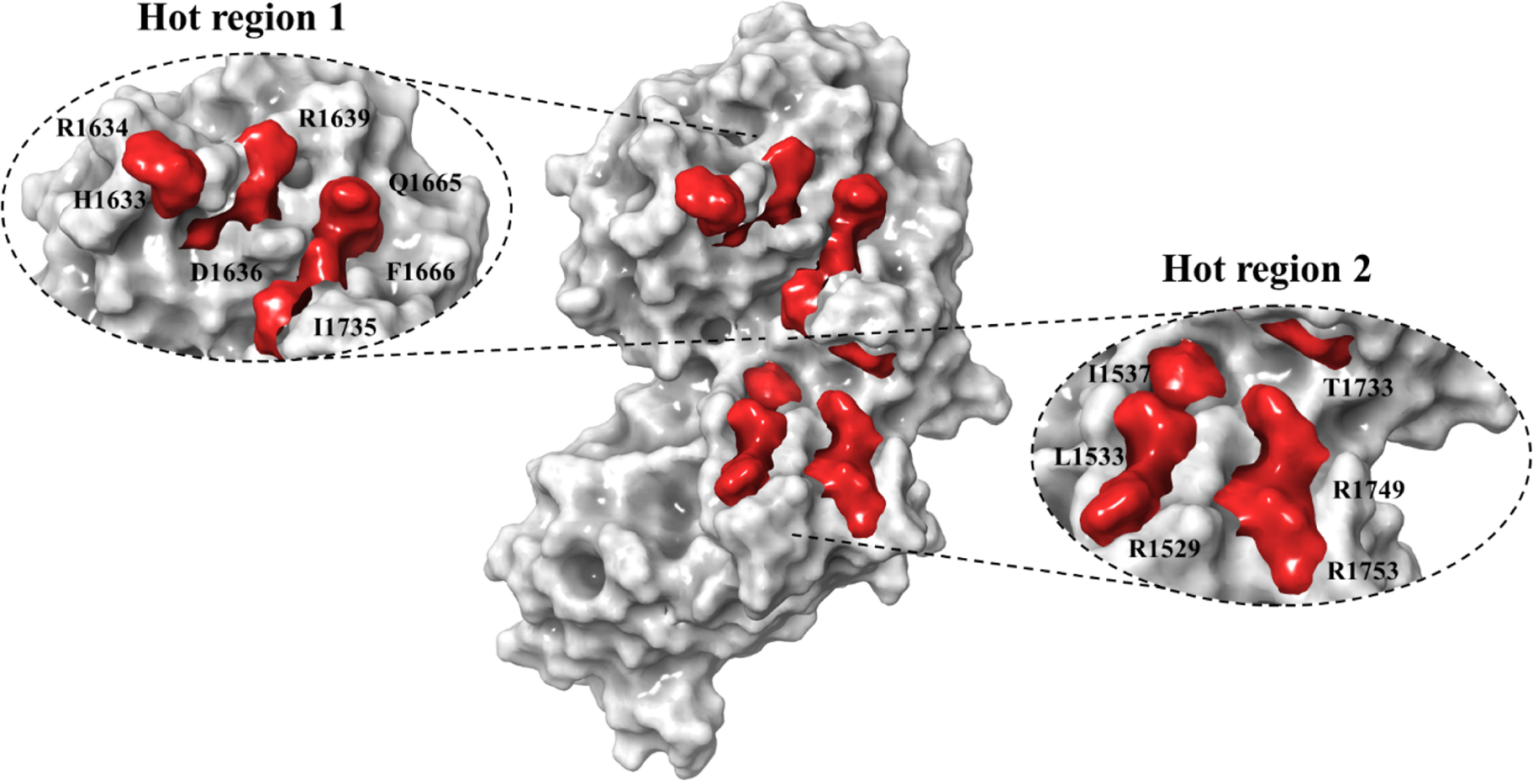
Surface representation of TSC2 model *Q3* showing two hot regions. Hot region 1 (catalytic site) includes residues H1633, R1634, D1636, R1639, Q1665, F1666, and I1735. Hot region 2 (recognition site) includes residues R1529, L1533, I1537, T1733, R1749, and R1753.

The major contributing residue type to the binding hotspots in TSC2 is arginine (R1529, R1634, R1639, R1749, and R1753), which is also in line with literature as it has been reported to be one of the common residues as binding hotspots.^59^ The arginine side chain is capable of forming both hydrogen bonds and salt bridges by its positively charged guanidinium head and van der Waals interactions since the body has hydrophobic character.^59^ The remaining residues at the interface are a mix of hydrophobic residues (L1533, I1537, F1666, T1733, and I1735) that form important van der Waals interactions, and polar residues (H1633, D1636, and Q1665) capable of forming hydrogen bonds. Binding hotspots are, in general, not evenly distributed over a protein–protein interface, but rather packed into hot regions.^60^ As evident from Figure 8, two clusters can be observed for TSC2 that are placed at the interface where Rheb can potentially bind; hot region 1, which is located around the catalytic helix (catalytic site), and hot region 2, which is located on the αC and αN helix (recognition site). Hot region 1 includes H1633, R1634, D1636, R1639, Q1665, F1666, and I1735, while hot region 2 comprises of R1529, L1533, I1537, R1749, and R1753.

The interaction tool in Schrödinger was used to predict noncovalent and π–π interactions between TSC2 and Rheb. The residues found to have interactions in model *Q3* after protein–protein docking are listed in Table 4. The interactions largely correlate with the binding hotspot residues calculated in Table 3. Hydrogen bond contacts were observed between the binding hotspot residues H1633^TSC2^–Y14^Rheb^, R1634^TSC2^–T88^Rheb^, D1636^TSC2^–R15^Rheb^, T1733^TSC2^–T73^Rheb^, and R1749^TSC2^–F70.

**Table 4:** Predicted Hydrogen Bonds and π–π Interactions between TSC2 and Rheb in Model *Q3*

Entry	TSC2	Rheb	Interaction	Distance (Å)
1	R1634	T88	H-bond	2.61
2	H1633	R15	Pi-stacking	5.69
3	H1633	Y14	H-bond	2.27
4	C1635	Y35	Aromatic H-bond	3.77
5	D1636	R15	H-bond	2.31
6	T1733	T73	H-bond	2.71
7	R1749	F70	H-bond	2.20

**Figure 9:**
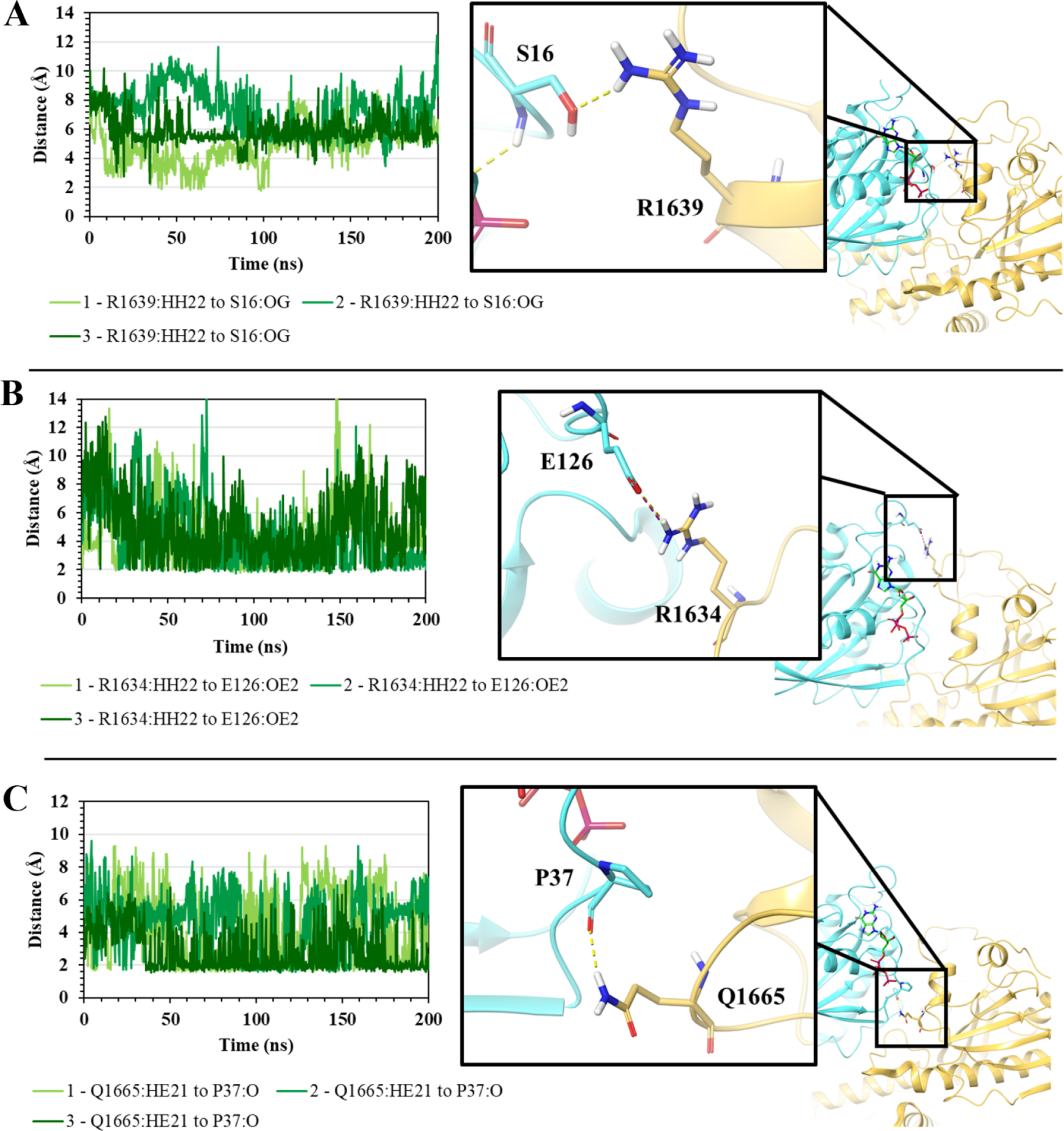
Distances between; (**A**) R1634^TSC2^ and E126^Rheb^ (**B**) R1639^TSC2^ and S16^Rheb^ (**C**) Q1665^TSC2^ and P37^Rheb^, and their cartoon representations of the corresponding interaction. Notation used is “Simulation no. - TSC2 residue & atom to Rheb residue & atom.”

The interactions of *Q3* from the triplicate MD simulations were further analyzed in order to obtain a more accurate estimate of the residues that play key roles in the binding of Rheb to TSC2. The evaluation was based on predicted noncovalent interactions and π–π interactions by the interaction panel of Schrödinger. Throughout the simulations, the interface residues of TSC2 and Rheb display a variety of interactions as a result of their conformational dynamics. Multiple interface residues exhibited H-bonding and/or salt bridges for at least 50% of the simulation time and are thus noted as stable interactions. As shown in Figure 9A, hotspot residue R1639^TSC2^ forms H-bonding with S16^Rheb^ with an average distance of 6.1 Å ± 1.3 Å over the three replicas. Hotspot residue R1634^TSC2^ with E126^Rheb^ forms H-bonding and salt bridges in all 3 replicas of the triplicate simulations with an average distance of 4.7 Å ± 2.2 Å (Figure 9B). Lastly, Q1665^TSC2^ forms H-bonding with the backbone of P37^Rheb^ (Figure 9C) with a distance of 3.9 Å ± 1.8 Å averaged over the three replicas. The remaining hotspot residues either lose their interactions as they turn away from the protein–protein interface or form interactions with various other residues over the course of the simulations. Previous studies have proven that residues G1595, G1596, D1636, H1640, and F1666 of TSC2 and residues R15, P37, I39, and D65 of Rheb are important for binding. Therefore, the interactions of these residues were further investigated in triplicate MD simulations. The analysis shows that, although in close proximity, no noncovalent or π–π interactions are preserved throughout the simulations. D1636^TSC2^ mostly interacts with residues on TSC2 instead of R15^Rheb^, while H1640^TSC2^ interacts with multiple residues of Rheb, including R15^Rheb^ and Y14^Rheb^ during the course of the simulations. R15 ^Rheb^ primarily forms salt bridges and H-bonds with D65^Rheb^. The hydrophilic region of F1666^TSC2^ stays near switch I of Rheb and, as mentioned above, forms H-bonds with the backbone of P37^Rheb^.

van der Waals interactions also contribute to the stability of the protein–protein complexes. As evidenced by the van der Waals energy contribution calculated by MM-GBSA in Section 3.3 of the triplicate MD simulations, one can conclude that these interactions are a crucial factor in the stability between the two protein structures and thus should not be ignored when analyzing the interactions at the protein–protein interface. Another factor to consider is the selectivity of TSC2-GAP for Rheb compared to other GTPases that TSC2 regulates, such as Rac1.^61^ Structural overlay of Rac1 with Rheb revealed that the residues critical for protein–protein interactions are not conserved in Rac1. We believe that the predicted binding mode and residues identified at the interface between TSC2 and Rheb can be employed as a guideline for inhibiting the TSC2–Rheb complex and identifying new inhibitors targeting MM.

**Figure 10:**
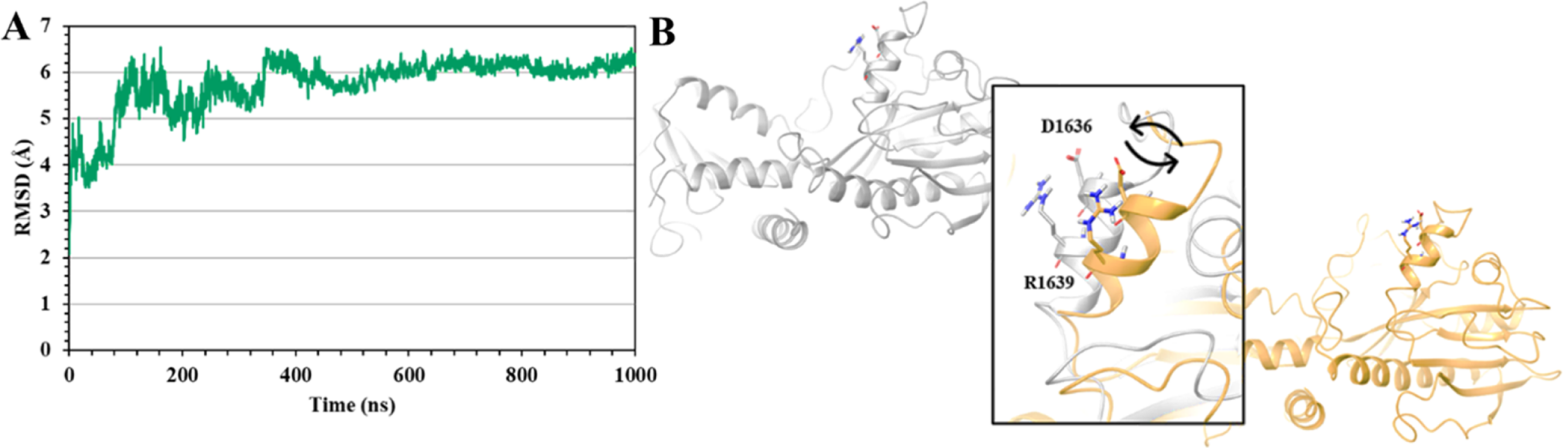
(**A**) α-carbon RMSD plot of the TSC2 protein structure *Q3* during the 1000 ns MD simulations. (**B**) Cartoon representations of the “breathing” motion of TSC2.

In addition to the above simulations, we carried out 1000 ns MD simulations of the TSC2 protein structure of the *Q3* model alone, in order to gain deeper insights into the dynamics of TSC2. This might be useful to further understand its binding with Rheb and potentially designing new inhibitors. An interesting aspect of the TSC2 was observed after simulating the protein structure, revealing that TSC2 exhibited a “breathing” motion of the catalytic helix throughout the 1000 ns simulation. This is demonstrated in Figure 10B, in which the “out” and “in” motion of the α3-helix is shown. In particular, the residues D1636 and R1639 seem to fluctuate notably and come back to their original position as a part of the “breathing” motion. Furthermore, the system appeared to be exceptionally stable after ∼360 ns with only minor fluctuations in the RMSD (Figure 10A). Here, the system stabilized in the more “open” structure, and the breathing motion, although still present, occurred less significantly.

Moreover, during the course of the 1000 ns simulation, the average RMSD of the TSC2 structure was found to be 6.5 Å, while the average RMSF (per residue) was calculated as 2.9 Å, both of which confirm a significant stability of the TSC2 model (*Q3*) used in this study. The structural transition between the “in” and “out” state, along with the converged TSC2 structure, could potentially generate the necessary gateway to specifically target TSC2 and inhibit the protein–protein interaction with Rheb.

## 4 Conclusions

The PI3K/AKT/mTORC1 pathway is one of the most deregulated pathways in human cancer. In MM, responses to stress such as hypoxia and proteasome inhibitors downregulate this pathway, which leads to the development of resistance toward proteasome inhibitors.^8^ The main contributor to the suppression of mTORC1 is TSC2, which is the GAP protein for Rheb. Under stress, TSC2 is released and inactivates Rheb, the key regulator of mTORC1, by catalyzing the hydrolysis of GTP to GDP. Previous research has shown that by reinstating mTORC1, the toxicity of PIs is reinstated in MM.^8^

In this computational study, we predict the putative binding mode of Rheb to TSC2 and obtain detailed knowledge of the key protein–protein interaction between the two. The TSC2-GAP region is the catalytic part of TSC2 onto which Rheb binds. Since TSC is a multiprotein complex, the protein chains that are not directly involved in binding were eliminated in order to reduce the computational time. By truncating the TSC complex in three distinct stages (*Q1*, *Q2,* and *Q3*), the most compatible model of the TSC2–Rheb complex was obtained. The TSC2–Rheb protein–protein complex was predicted with high accuracy as a result of a robust consensus docking strategy. It was further concluded from the MD simulations and MM-GBSA analysis that the structure *Q3* has strong interactions and highest stability relative to *Q1* and *Q2*, with an average binding free energy of −106 kcal/mol. The stability of *Q3* was further validated by triplicate MD simulations, which provided a thorough understanding of the binding mode and key protein–protein interactions occurring between TSC2 and Rheb. It was established that N1643^TSC2^ is at an acceptable distance to the γ-phosphate group of GTP (in the presence of a water molecule) throughout the simulations and confirms the involvement of this asparagine thumb in the hydrolysis of GTP to GDP at the catalytic binding site of TSC2 by locking a water molecule in place. Insights into the protein–protein interactions were further provided by hotspot analysis. Here, it was evidenced that the major contribution of TSC2 residues binding to Rheb is centered at two main regions: region 1 (H1633, R1634, D1636, R1639, Q1665, F1666, and I1735) located at the catalytic binding site, and region 2 (R1529, L1533, I1537, T1733, R1749, and R1753) located at the recognition site. By triplicate MD simulations, it was demonstrated that residues R1634^TSC2^–E126^Rheb^, R1639^TSC2^–S16^Rheb^, and Q1665^TSC2^–P37^Rheb^ interact by H-bonding or salt bridges throughout the simulations. MM-GBSA calculations of the trajectories furthermore demonstrated that van der Waals interactions between residues play a crucial role in binding. Together with the residues found to be important through mutational studies of the catalytic mechanism^12,22,23^ i.e., N1643, D1636, H1640, and F1666 on TSC2, a clear target site could be envisioned for the design and screening of small molecules inhibiting the TSC2–Rheb complex.

Targeting the residues responsible for the major binding interaction at the catalytic site paves the way for the development and optimization of new drug-like molecules disrupting the TSC2 – Rheb binding and catalytic reaction. The outcomes of this work are expected to benefit the development of adjuvant inhibitors, which is a promising strategy to activate mTORC1 and reinstate the efficacy of PIs targeting multiple myeloma.

## Supporting Information Available

Number of poses selected from each docking engine for refinement of each of the three TSC2–Rheb complexes, the three TSC2–Rheb docked models *Q1*, *Q2*, and *Q3*, contribution of each docking engine in the most populated clusters of the TSC2–Rheb complexes, the model of TSC2-GAP predicted by Hansmann et al.,^12^ superposition of *Q1*, *Q2*, and *Q3*, structure alignment of first and last frame from the triplicate MD simulations of *Q3*, and MM-GBSA binding free energy plot from the triplicate MD simulations of *Q3* (PDF)
