# Supplementary material for: Unraveling the Binding Mode of TSC2–Rheb through Protein Docking and Simulations
Source: Biochemistry. 2025 Feb 13;64(5):1006–19. doi: 10.1021/acs.biochem.4c00562 (PMC11883811; doi:10.1021/acs.biochem.4c00562)
Supplement: Supplementary file 1 — bi4c00562_si_001.pdf [file bi4c00562_si_001.pdf]

Supporting information

Unravelling the binding mode of TSC2-Rheb through protein docking and simulations

Berith F. Pape[1], Shraddha Parate[1], Leif A. Eriksson[1]* and Vibhu Jha[1,2]*

[1]Department of Chemistry and Molecular Biology, University of Gothenburg, 405 30 Göteborg, Sweden

[2]Institute of Cancer Therapeutics, School of Pharmacy and Medical Sciences, Faculty of Life Sciences, University of Bradford, Bradford, UK BD71DP

Correspondence:

*Leif A. Eriksson, Department of Chemistry and Molecular Biology,

University of Gothenburg, 405 30 Göteborg, Sweden.

Email: leif.eriksson@chem.gu.se

Phone: +46 317869117

*Vibhu Jha, Institute of Cancer Therapeutics, School of Pharmacy and Medical Sciences,

Faculty of Life Sciences, University of Bradford, Bradford, UK BD71DP

Email: v.jha2@bradford.ac.uk

Phone: +447785688003

Supplementary table and figures

Figure S1. Cartoon representation of TSC2-GAP region with close up on the active site. P2

Figure S2. Cartoon representation of Rheb with close up on the active site. P2

Table S1. The number of poses selected from each docking engine for refinement of each TSC2-Rheb complex. P3

Figure S3. Cartoon representation of the three TSC2-Rheb docked models *Q1*, *Q2* and *Q3*. P3

Figure S4. The contribution of each docking engines in the most populated clusters of the TSC2-Rheb complexes 1, 2 and 3. P4

Figure S5. Superposition of the three models *Q1*, *Q2* and *Q3*. P4

Figure S6. The model of TSC2-GAP predicted by Hansmann et al. P4

Figure S7. MM-GBSA binding free energy plot from the triplicate MD simulations of the TSC2-Rheb model *Q3*. P5

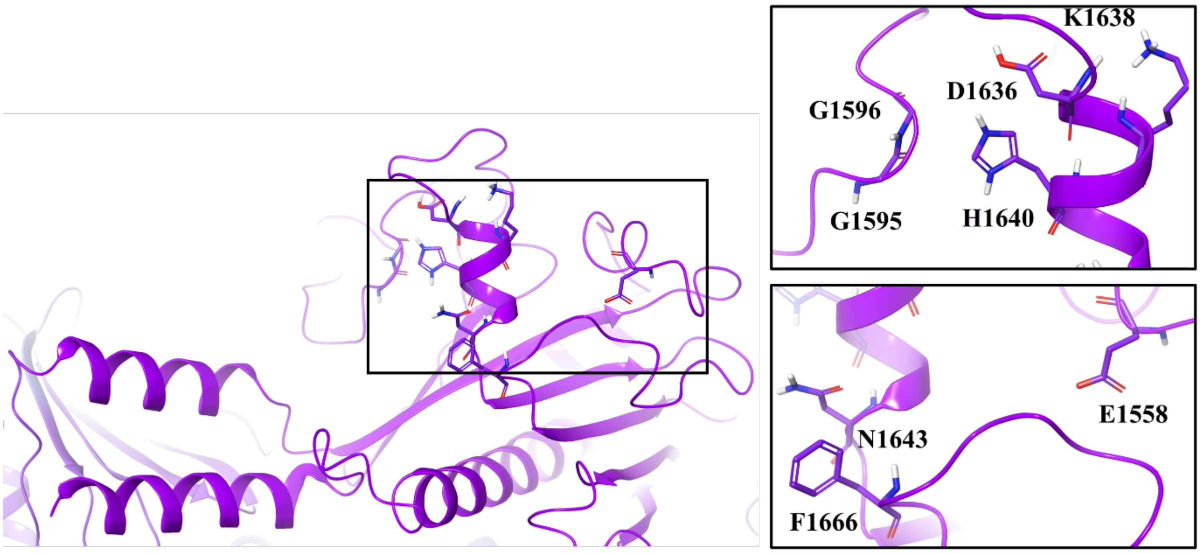

Figure S1. Cartoon representation of TSC2-GAP region with close up on the active site, residues E1558, G1595, G1596, D1636, H1640, N1643 and F1666 shown as sticks.

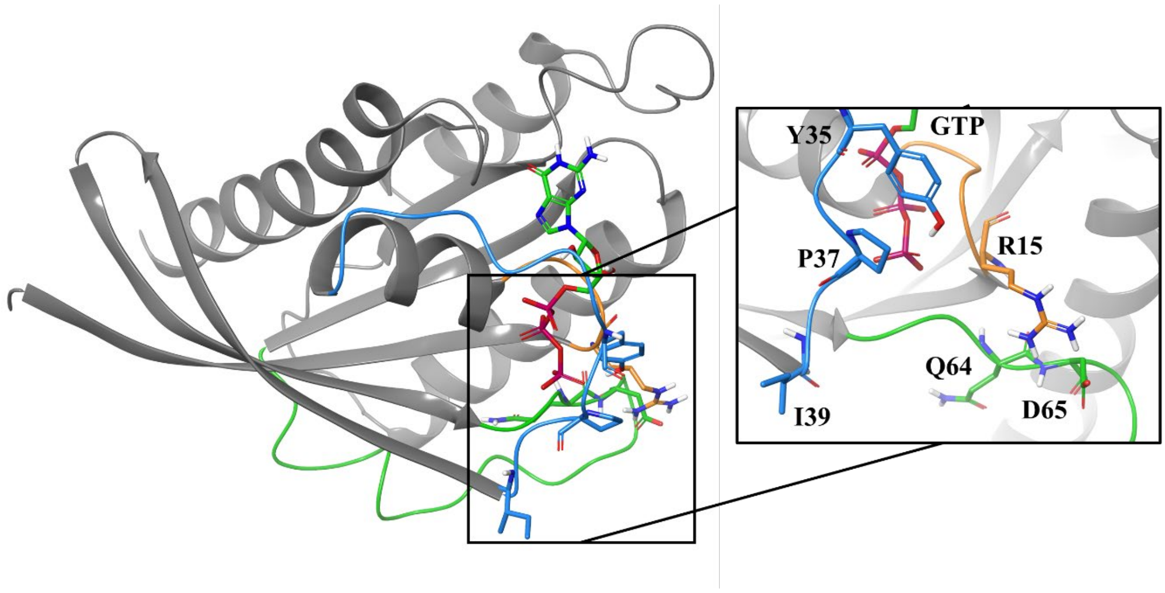

Figure S2. Cartoon representation of Rheb with close up on the active site, residues R15, Y35, P37, I39, Q65, D65 and GTP shown as sticks.

Table S1: The number of poses selected from each docking engine for refinement of each TSC2-Rheb complex.

**TSC2-Rheb complex 1**		**TSC2-Rheb complex 2**		**TSC2-Rheb complex 3**	
**Docking engine**		**Docking engine**		**Docking engine**	
Schrödinger	10	Schrödinger	7	Schrödinger	8
MOE	10	MOE	10	MOE	10
HDOCK	10	HDOCK	10	HDOCK	10
HADDOCK	10	HADDOCK	10	HADDOCK	7
GRAMM	-	GRAMM	10	GRAMM	6
ZDOCK	4	ZDOCK	2	ZDOCK	10
ClusPro	10	ClusPro	7	ClusPro	2
PyDock	10	PyDock	7	PyDock	5
LZerD	10	LZerD	3	LZerD	3
Total	74	Total	66	Total	61
After Refinements	370	*After Refinements*	330	*After Refinements*	305

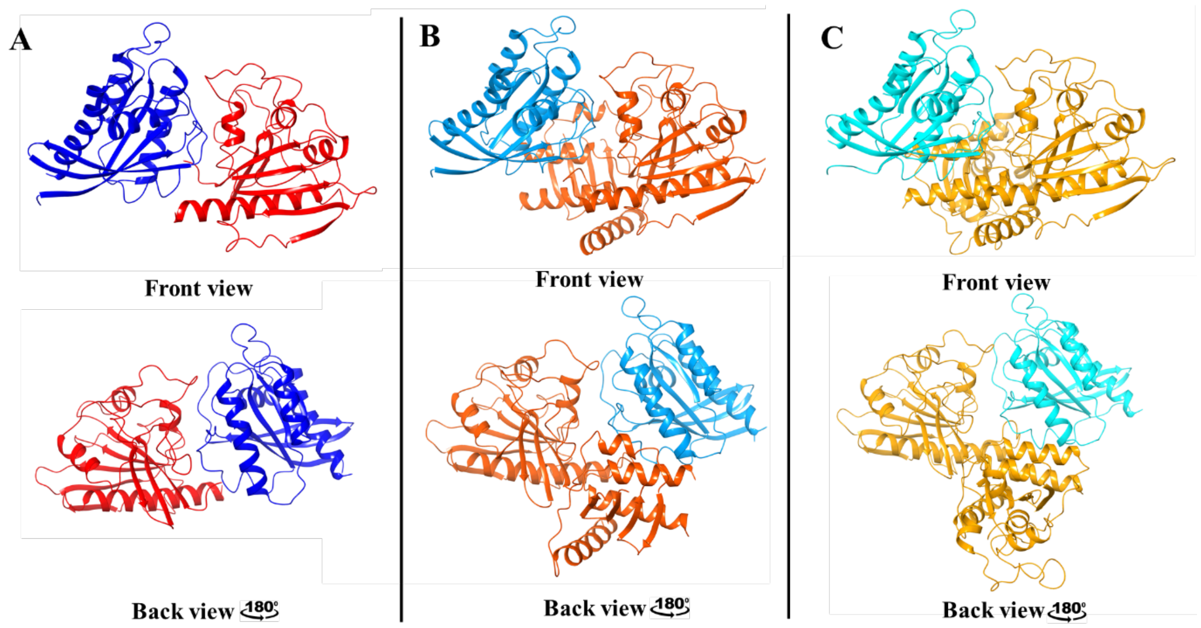

Figure S3. Cartoon representation of **(A)** model *Q1* with TSC2 (red) and Rheb (navy blue), **(B)** model *Q2* with TSC2 (orange) and Rheb (aqua) and **(C)** model *Q3* with TSC2 (yellow) and Rheb (turquoise).

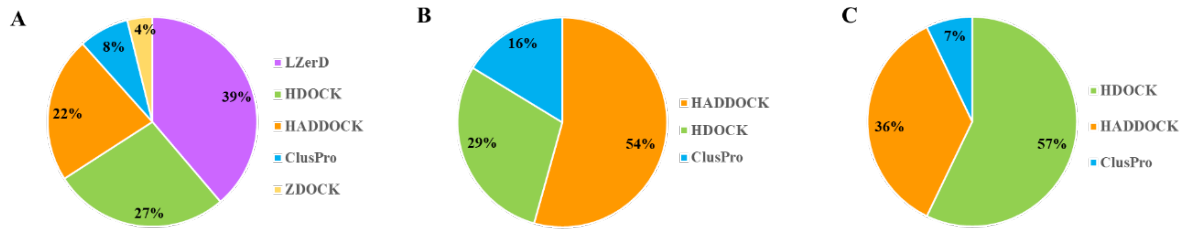

Figure S4. The contribution of each docking engines in the most populated clusters of **(A)** TSC2-Rheb complex 1 **(B)** TSC2-Rheb complex 2 and **(C)** TSC2-Rheb complex 3.

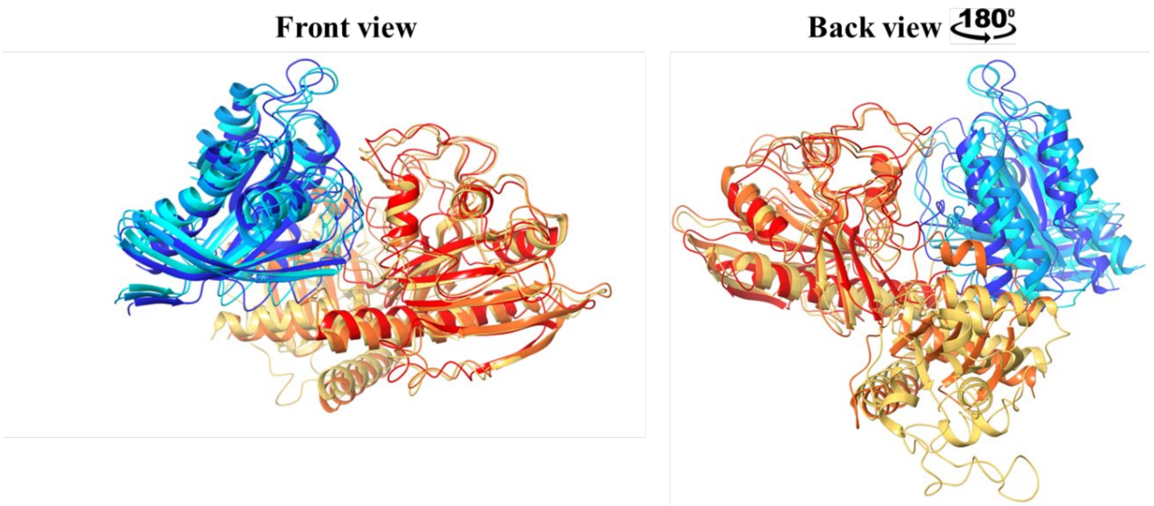

Figure S5. Superposition of *Q1* (TSC2: red, Rheb: navy blue), *Q2* (TSC2: orange, Rheb: aqua) and *Q3* (TSC2: yellow, Rheb: turquoise) front and back view.

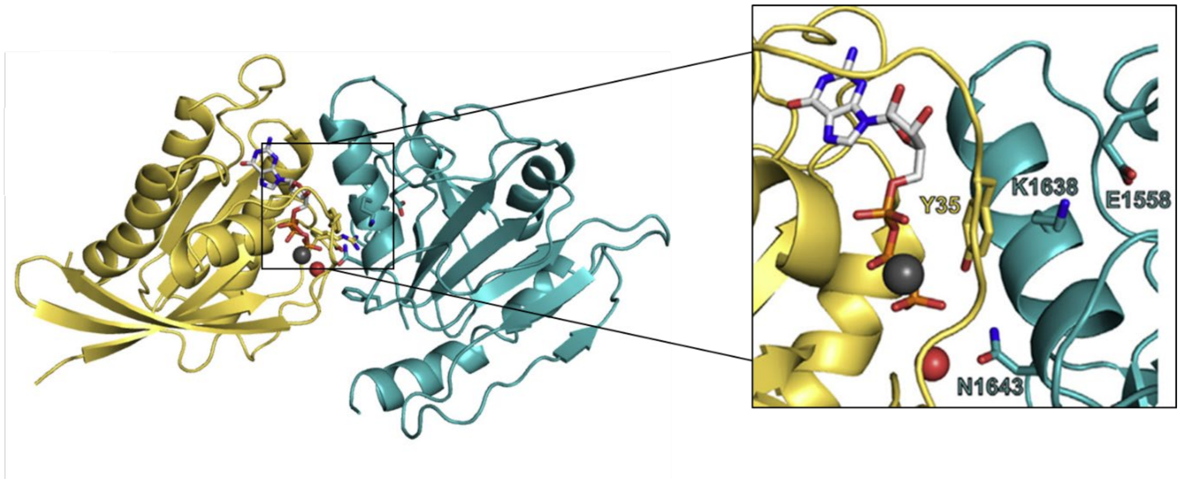

Figure S6. The model of TSC2-GAP (cyan) - Rheb (yellow) predicted by Hansmann et al., *Structure* **28** (2020) 933-942.e4.

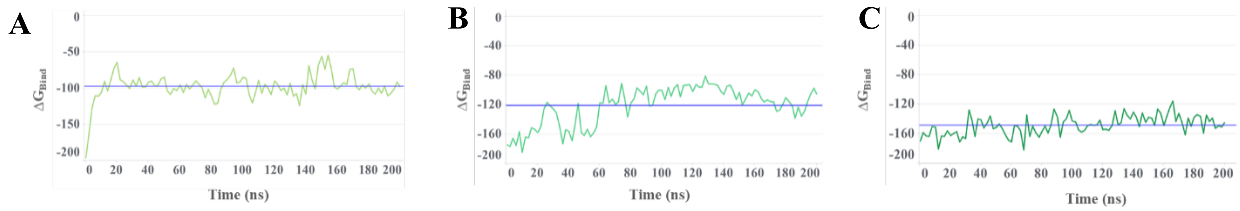

Figure S7. MM-GBSA binding free energy plot (kcal/mol) from triplicate MD simulations of *Q3*. The averaged values are represented by a blue line (A) Replica 1 (B) Replica 2 and (C) Replica 3.
